# Added Value of Medical Subject Headings Terms in Search Strategies of Systematic Reviews: Comparative Study

**DOI:** 10.2196/53781

**Published:** 2024-11-19

**Authors:** Victor Leblanc, Aghiles Hamroun, Raphaël Bentegeac, Bastien Le Guellec, Rémi Lenain, Emmanuel Chazard

**Affiliations:** 1 Public Health Department CHU Lille Université de Lille Lille France; 2 ULR 2694 Metrics, CERIM Université de Lille Lille France

**Keywords:** Medical Subject Headings, MeSH, MeSH thesaurus, systematic review, PubMed, search strategy, comparative analysis, literature review, positive predictive value, PPV, review, scientific knowledge, medical knowledge, utility, systematic literature review

## Abstract

**Background:**

The massive increase in the number of published scientific articles enhances knowledge but makes it more complicated to summarize results. The Medical Subject Headings (MeSH) thesaurus was created in the mid-20th century with the aim of systematizing article indexing and facilitating their retrieval. Despite the advent of search engines, few studies have questioned the relevance of the MeSH thesaurus, and none have done so systematically.

**Objective:**

The objective of this study was to estimate the added value of using MeSH terms in PubMed queries for systematic reviews (SRs).

**Methods:**

SRs published in 4 high-impact medical journals in general medicine over the past 10 years were selected. Only SRs for which a PubMed query was provided were included. Each query was transformed to obtain 3 versions: the original query (V1), the query with free-text terms only (V2), and the query with MeSH terms only (V3). These 3 queries were compared with each other based on their sensitivity and positive predictive values.

**Results:**

In total, 59 SRs were included. The suppression of MeSH terms had an impact on the number of relevant articles retrieved for 24 (41%) out of 59 SRs. The median (IQR) sensitivities of queries V1 and V2 were 77.8% (62.1%-95.2%) and 71.4% (42.6%-90%), respectively. V1 queries provided an average of 2.62 additional relevant papers per SR compared with V2 queries. However, an additional 820.29 papers had to be screened. The cost of screening an additional collected paper was therefore 313.09, which was slightly more than triple the mean reading cost associated with V2 queries (88.67).

**Conclusions:**

Our results revealed that removing MeSH terms from a query decreases sensitivity while slightly increasing the positive predictive value. Queries containing both MeSH and free-text terms yielded more relevant articles but required screening many additional papers. Despite this additional workload, MeSH terms remain indispensable for SRs.

## Introduction

The number of articles published in scientific and medical journals have been increasing exponentially since the late 20th century. In 2021 alone, over 1,700,000 indexed, full-text articles were included in the PubMed database. In response to the massive production of scientific knowledge, the need for access to synthetic scientific data has been driven by the emergence of evidence-based medicine [1] and the establishment of national regulatory bodies, medical associations, and learned societies that provide guidelines on best practice.

In this context, systematic reviews (SRs; a type of analysis developed in the 1970s) are becoming more important. Given that quality of an SR depends largely on the research methodology, building search queries is a crucial part of the review process. The challenge of constructing a query for a SR lies in the absolute necessity of being as sensitive as possible, despite the fact that this query will return at most a few tens of thousands of articles among the hundreds of millions that make up the scientific literature [2].

In the mid-20th century, researchers started to develop a common vocabulary that facilitated article indexing and retrieval and helped to avoid misunderstandings [3-5]. These efforts led to the creation of the Medical Subject Headings (MeSH) thesaurus in the 1960s by the US National Library of Medicine (NLM) [6]. PubMed (the NLM’s search engine), which is one of the most widely used search engines [7], heavily relies on MeSH terms to assist users in their literature searches. The MeSH thesaurus is intended to facilitate literature searches by limiting term permutations [8,9]. In other words, it assigns a unique term to a concept—regardless of the language used or the time period concerned.

Subsequent improvements in search engine performance have enabled researchers to query databases with simple free-text terms, rather than MeSH terms. Furthermore, the massive influx of publications and the emergence of many new scientific and medical topics have led to delays in MeSH indexing and difficulties in updating the thesaurus [10]. In addition, although frequently recommended [11-13], the value of using the MeSH thesaurus in queries for literature reviews has never been systematically assessed. The few studies to have tested the utility of MeSH terms in SRs have limitations, such as a small sample size or a lack of generalizability [14-20]. Finally, some studies simply compared the numbers of results retrieved for a given query but did not evaluate the results’ relevance [21].

To the best of our knowledge, only 1 study has extensively explored the relevance of MeSH terms with regard to the results of SRs [22]. The study concluded that the use of queries based on free-text alone (ie, free-text terms) appeared to decrease the retrieval of articles of interest, relative to queries based on both free-text terms and MeSH terms. However, this study included SRs from a single research center, which limited the generalizability of the findings. Furthermore, the MEDLINE database was queried with the Ovid search engine, rather than PubMed. We therefore decided to evaluate this question in more detail. The objective of this work was to estimate the added value of using MeSH terms in PubMed queries for SRs.

## Methods

### Paper Selection

We first selected the top 6 journals in the “Medicine. General & Internal” Journal Citation Reports category, according to the impact factors computed by Clarivate [23,24]. Next, we selected all the PubMed-indexed SRs published in the 6 journals between 2012 and 2021 and for which the free full text was available on PubMed Central. The time period was chosen arbitrarily, with the objective of obtaining at least 60 SRs. The following PubMed query was used: ‘(“The New England Journal of Medicine”[Journal] OR “Lancet London England”[Journal] OR “JAMA”[Journal] OR “Nature Reviews Disease Primers”[Journal] OR “BMJ Clinical Research Ed”[Journal] OR “Annals of Internal Medicine”[Journal]) AND “loattrfree full text”[Filter] AND 2012/01/01:2021/12/31[Date - Publication] AND systematic review[Filter]’.

The exclusion criteria were as follows: (1) articles other than an SR, (2) the absence of a published search query, (3) the use of queries in multiple parts that had to be assembled, (4) the absence of a query specifically built for PubMed, (5) a query that did not return any results, (6) a query that returned more than 100,000 results, and (7) a query with only MeSH terms or without MeSH terms. The sorting was carried out by a single researcher (VL).

### Analysis of the PubMed Results

**Figure 1:**
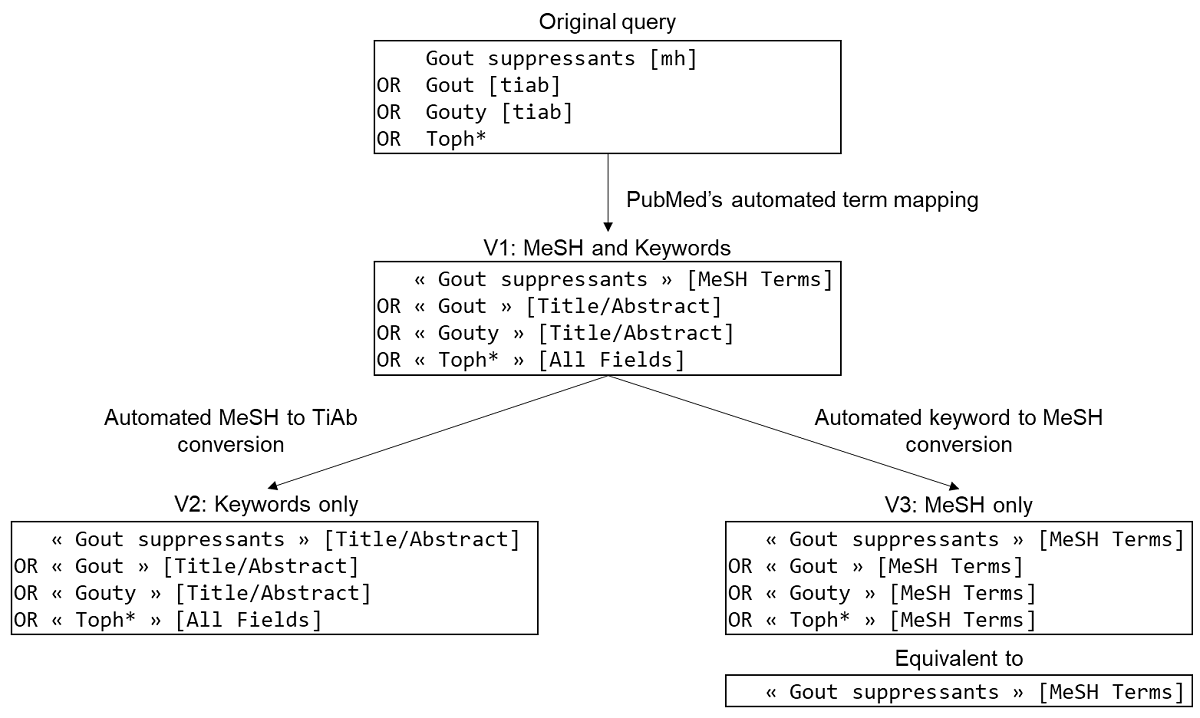
Example of an automated transformation of queries. MeSH: Medical Subject Headings.

The query was extracted from each included SR and inserted into the PubMed search bar. PubMed has a feature called automatic term mapping (ATM) [25]; when terms not enclosed in quotation marks are inserted in the search bar, they are automatically transformed into a query segment that contains several descriptors, such as [MeSH terms], [tiab], and [all fields]. To ensure greater reproducibility, we checked for the automatic transformation of queries. This step was important because PubMed’s ATM feature might add MeSH terms to query initially considered to be free of such terms. Hence, we always retrieved the query formatted by PubMed’s ATM (henceforth referred to as V1; Figure 1).

For each SR, V1 was transformed into a V2 query by replacing each MeSH term in the query with a free-text term that had to be present in the title or in the abstract. To do this, we simply replaced the [MeSH] tag with a [Title/Abstract] tag. Hence, the resulting V2 did not contain any explicit [MeSH] tags (Figure 1). Lastly, the V3 (MeSH-only) query was obtained by transforming all free-text terms into MeSH terms. It should be noted that terms stated as MeSH terms in the query but that do not actually exist in the MeSH thesaurus are ignored by the PubMed engine; this is equivalent to deleting the terms (Figure 1 [26]).

The transformations from V1 to V2 and V3 were the same for all queries, regardless of whether they contained MeSH terms only or free-text terms only. However, we noted that some PubMed filters are based on MeSH terms [27]. It would therefore not be relevant to convert these terms into free-text terms. We drew up a list of these terms so that they were not transformed and were still able to serve as filters. Those 14 terms are “80 and over,” “adolescent,” “adult,” “aged,” “animals,” “child,” “female,” “humans,” “infant,” “male,” “middle aged,” “newborn,” “preschool,” and “young adult.”

Hence, each SR had a query written by the SR’s authors (a combination of MeSH and free-text terms; V1), a free-text-only query (V2), and a MeSH-only query (V3). Therefore, we intend to interpret the comparison of V2 with V1 as the added value of MeSH terms, and we intend to interpret the comparison of V3 with V1 as the added value of free-text terms.

Each query was submitted to the PubMed search engine, and the results were retrieved and sorted by the “Best Match” option. If there were more than 10,000 results, only the first 10,000 results were retained; in fact, PubMed does not allow more than 10,000 results to be extracted. The results were identified by their PubMed Identifier (PMID).

For each SR, the “gold standard” (GS) consisted of the articles selected by the authors of the SR. Each SR was read in order to extract the list of PMIDs selected by the authors. This work was done “by hand” by 4 researchers (VL, RB, BLG, and AH). Publications cited in the SR but not indexed in MEDLINE were not analyzed. If the reference section did not contain the items selected in the SR, data extraction from supplementary files allowed for the completion of the GS.

### Data Analysis

For each SR, we obtained 4 lists of PMIDs: the GS, those retrieved by V1 (MeSH and free-text terms), those retrieved by V2 (free-text terms only), and those retrieved by V3 (MeSH terms only). For each list, we computed the sensitivity (also referred to as “recall”) and the positive predictive value (PPV; also referred to as “precision”) with respect to the GS. We then computed the *F*_1_-score, which is the harmonic mean of the sensitivity and the PPV.

For each query *i* (V1, V2, and V3), the odds for the PPV was defined as the ratio between 2 numbers:



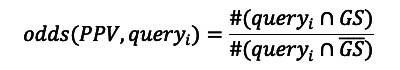



Next, for a given SR and using the same GS, the odds ratio (OR) of query_2_ to query_1_ for the PPV was defined as:



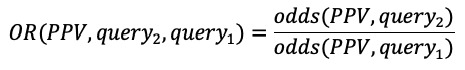



Likewise, the odds for the sensitivity of each query *i* (V1, V2, and V3) was defined as the ratio between 2 numbers:



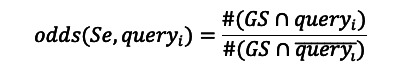



Hence, for a given SR and using the same GS, the OR for query_2_ versus query_1_ with regard to sensitivity was:



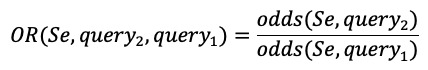



We computed the respective ORs for V2 versus V1 and V3 versus V1 for the PPV and the sensitivity:



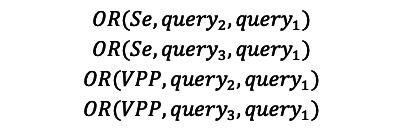



An OR of 1 means that the queries have the same level of performance with regard to the chosen indicator. An OR<1 denotes worse performance, and an OR>1 denotes better performance.

### Statistical Analysis

Qualitative variables, binary variables, or discrete variables with very few modalities were expressed as the frequency (percentage). Quantitative variables were expressed as the mean (SD) when symmetrically distributed and the median (IQR) when not. The independence of 2 qualitative variables was probed in a chi-square test.

All statistical tests were 2-sided. The threshold for statistical significance was set to *P*<.05. The 95% CI of a proportion was calculated using the Wald method. Statistical analyses were performed with R software (R Core Team), RStudio software (Posit PBC), and the R *metafor* package [28-30].

### Ethical Considerations

The research was performed using publicly available documents. It did not involve individuals or personal data. Approval by an institutional review board was not required.

## Results

### Flowchart

**Figure 2:**
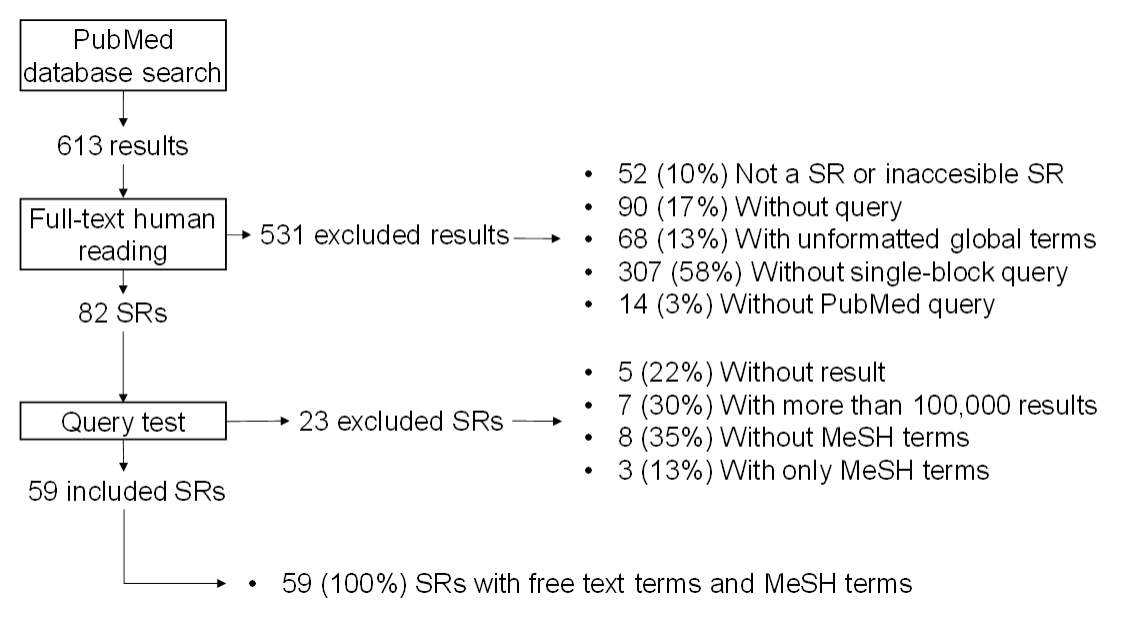
Flowchart for the selection of systematic reviews. MeSH: Medical Subject Heading; SR: systematic review.

The SRs used to compile the set of queries were selected by a single researcher (VL; Figure 2).

### Description of the Included Systematic Reviews

A total of 59 SRs were selected for analysis, which contained both MeSH terms and free-text terms (Table 1 and Multimedia Appendices 1 and 2 [26,31-88]).

**Table 1:** General description of the attributes for each included systematic review.

SR’s^a^ PMID^b^	Items, n					Number of GS found				V1^c^				V2^d^				V3^e^		
	V1	V2	V3	GS^f^	V1		V2	V3	Se^g^		PPV^h^	*F*-Sc^i^	Se		PPV	*F*-Sc	Se		PPV	F-Sc
33472813 [46]	297	294	147	30	27		27	24	0.900		0.091	0.165	0.900		0.092	0.167	0.800		0.163	0.271
33441384 [50]	4408	2265	901	16	14		13	11	0.875		0.003	0.006	0.812		0.006	0.011	0.688		0.012	0.024
33186535 [49]	145	153	1	6	4		4	1	0.667		0.028	0.053	0.667		0.026	0.050	0.167		1.000	0.286
33148618 [40]	9024	10000	3706	66	16		18	3	0.242		0.002	0.004	0.273		0.002	0.004	0.045		0.001	0.002
32909814 [59]	349	349	40	9	1		1	0	0.111		0.003	0.006	0.111		0.003	0.006	0.000		0.000	0.000
32496521 [47]	94	162	0	24	0		0	0	0.000		0.000	0.000	0.000		0.000	0.000	0.000		0.000	0.000
32459529 [54]	1950	812	1441	9	7		2	7	0.778		0.004	0.007	0.222		0.002	0.005	0.778		0.005	0.010
32442035 [73]	10000	10000	842	15	8		7	2	0.533		0.001	0.002	0.467		0.001	0.001	0.133		0.002	0.005
32371466 [64]	4185	3320	324	50	49		48	21	0.980		0.012	0.023	0.960		0.014	0.028	0.420		0.065	0.112
32199484 [70]	6347	6111	1231	128	0		0	0	0.000		0.000	0.000	0.000		0.000	0.000	0.000		0.000	0.000
31255301 [43]	1164	1023	456	61	41		35	34	0.672		0.035	0.067	0.574		0.034	0.065	0.557		0.075	0.132
30884526 [33]	770	716	30	79	0		0	0	0.000		0.000	0.000	0.000		0.000	0.000	0.000		0.000	0.000
30617123 [75]	1086	788	292	29	28		26	14	0.966		0.026	0.050	0.897		0.033	0.064	0.483		0.048	0.087
30326495 [77]	4360	2212	951	158	123		117	62	0.778		0.028	0.054	0.741		0.053	0.099	0.392		0.065	0.112
30158148 [41]	10000	2673	395	45	33		24	28	0.733		0.003	0.007	0.533		0.009	0.018	0.622		0.071	0.127
29049756 [80]	3155	2858	913	20	16		16	2	0.800		0.005	0.010	0.800		0.006	0.011	0.100		0.002	0.004
28903922 [35]	687	610	95	24	23		23	16	0.958		0.033	0.065	0.958		0.038	0.073	0.667		0.168	0.269
27893131 [60]	10000	10000	2439	48	34		33	27	0.708		0.003	0.007	0.688		0.003	0.007	0.562		0.011	0.022
27802505 [74]	2299	2227	195	21	19		19	14	0.905		0.008	0.016	0.905		0.009	0.017	0.667		0.072	0.130
27802478 [26]	3847	3525	2526	89	45		43	43	0.506		0.012	0.023	0.483		0.012	0.024	0.483		0.017	0.033
27548070 [63]	1634	910	626	26	20		10	9	0.769		0.012	0.024	0.385		0.011	0.021	0.346		0.014	0.028
27142267 [78]	10000	10000	10000	10	7		9	5	0.700		0.001	0.001	0.900		0.001	0.002	0.500		0.000	0.001
26903336 [81]	903	414	115	92	47		47	0	0.511		0.052	0.094	0.511		0.114	0.186	0.000		0.000	0.000
26349907 [53]	2675	265	1728	8	0		0	0	0.000		0.000	0.000	0.000		0.000	0.000	0.000		0.000	0.000
26199070 [58]	554	303	0	20	17		15	0	0.850		0.031	0.059	0.750		0.050	0.093	0.000		0.000	0.000
26109551 [66]	298	297	14	14	9		9	5	0.643		0.030	0.058	0.643		0.030	0.058	0.357		0.357	0.357
25770113 [42]	3046	2761	1956	7	7		5	7	1.000		0.002	0.005	0.714		0.002	0.004	1.000		0.004	0.007
25569206 [39]	746	691	0	49	49		49	0	1.000		0.066	0.123	1.000		0.071	0.132	0.000		0.000	0.000
25556126 [67]	212	206	50	9	8		8	4	0.889		0.038	0.072	0.889		0.039	0.074	0.444		0.080	0.136
25006006 [52]	834	395	407	25	24		23	20	0.960		0.029	0.056	0.920		0.058	0.110	0.800		0.049	0.093
24727842 [62]	2046	1989	0	69	66		66	0	0.957		0.032	0.062	0.957		0.033	0.064	0.000		0.000	0.000
24157497 [87]	10000	81	8636	61	61		0	61	1.000		0.006	0.012	0.000		0.000	0.000	1.000		0.007	0.014
24046285 [48]	978	978	812	12	12		12	12	1.000		0.012	0.024	1.000		0.012	0.024	1.000		0.015	0.029
23935058 [69]	628	20	887	5	3		0	1	0.600		0.005	0.009	0.000		0.000	0.000	0.200		0.001	0.002
23900314 [51]	499	300	195	6	5		5	4	0.833		0.010	0.020	0.833		0.017	0.033	0.667		0.021	0.040
23529983 [65]	283	160	0	8	8		2	0	1.000		0.028	0.055	0.250		0.013	0.024	0.000		0.000	0.000
23420235 [37]	6848	2305	3434	27	25		20	10	0.926		0.004	0.007	0.741		0.009	0.017	0.370		0.003	0.006
23033409 [86]	434	258	0	16	11		11	0	0.688		0.025	0.049	0.688		0.043	0.080	0.000		0.000	0.000
22986378 [76]	8633	7169	3884	40	26		38	19	0.650		0.003	0.006	0.950		0.005	0.011	0.475		0.005	0.010
22422870 [55]	1010	980	0	4	3		3	0	0.750		0.003	0.006	0.750		0.003	0.006	0.000		0.000	0.000
22323502 [32]	10000	10000	9784	5	3		3	2	0.600		0.000	0.001	0.600		0.000	0.001	0.400		0.000	0.000
22226047 [44]	1911	1239	1807	49	38		32	35	0.776		0.020	0.039	0.653		0.026	0.050	0.714		0.019	0.038
33176180 [84]	87	87	0	4	3		3	0	0.750		0.034	0.066	0.750		0.034	0.066	0.000		0.000	0.000
32479176 [68]	303	266	64	29	28		26	2	1.000		0.092	0.169	0.931		0.098	0.177	0.069		0.031	0.043
32427305 [57]	2035	2004	0	13	12		12	0	0.923		0.006	0.012	0.923		0.006	0.012	0.000		0.000	0.000
31727627 [56]	1884	1878	610	132	127		127	29	0.985		0.067	0.126	0.985		0.068	0.127	0.227		0.048	0.079
31585960 [36]	10000	10000	9514	227	186		206	139	0.819		0.019	0.036	0.907		0.021	0.040	0.612		0.015	0.029
30383109 [72]	4723	4716	570	38	36		36	2	0.947		0.008	0.015	0.947		0.008	0.015	0.053		0.004	0.007
28348110 [79]	27	27	0	24	1		1	0	0.042		0.037	0.039	0.042		0.037	0.039	0.000		0.000	0.000
28114600 [71]	85	35	25	68	9		3	0	0.132		0.106	0.118	0.044		0.086	0.058	0.000		0.000	0.000
26868137 [34]	10000	10000	10000	32	2		7	2	0.062		0.000	0.000	0.219		0.001	0.001	0.062		0.000	0.000
26830221 [82]	6167	1166	5102	76	74		28	72	0.974		0.012	0.024	0.368		0.024	0.045	0.947		0.014	0.028
26830055 [45]	6167	1166	5102	29	29		19	27	1.000		0.005	0.009	0.655		0.016	0.032	0.931		0.005	0.011
26420598 [83]	8405	8387	2421	57	47		47	30	0.825		0.006	0.011	0.825		0.006	0.011	0.526		0.012	0.024
26420387 [38]	8405	8387	2421	78	59		59	30	0.756		0.007	0.014	0.756		0.007	0.014	0.385		0.012	0.024
25059938 [61]	1524	1503	33	21	14		14	1	0.667		0.009	0.018	0.667		0.009	0.018	0.048		0.030	0.037
24592495 [31]	873	798	100	16	9		9	0	0.562		0.010	0.020	0.562		0.011	0.022	0.000		0.000	0.000
23460092 [85]	2426	2411	0	20	18		18	0	0.900		0.007	0.015	0.900		0.007	0.015	0.000		0.000	0.000
22777524 [88]	4645	3048	2838	37	37		35	31	1.000		0.008	0.016	0.946		0.011	0.023	0.838		0.011	0.022

^a^SR: systematic review.

^b^PMID: PubMed Identifier.

^c^V1: original query.

^d^V2: query with free-text terms only.

^e^V3: query with Medical Subject Headings terms only.

^f^GS: gold standard.

^g^Se: sensitivity.

^h^PPV: positive predictive value.

^i^*F*-Sc: *F*_1_-score.

Of the 59 selected SRs, 29 (49%) came from *The BMJ*, 19 (32%) came from the *Annals of Internal Medicine*, 6 (10%) came from *The Lancet*, and 5 (9%) came from the *Journal of the American Medical Association*. The publication dates were evenly distributed; the mean publication year and the median publication year were both 2016.

The countries of origin of the first authors were available for 49 (83%) SRs. The 3 most frequent countries of origin were the United States (21/49, 43%), the United Kingdom (5/49, 10%), and Canada (5/49, 10%).

### Quantification of the Utility of Medical Subject Headings Terms

The queries contained a median (IQR) of 43 (17.0-98) terms. The median (IQR) number of MeSH terms in the V1 queries was 6.0 (3.0-19.5). The median (IQR) proportion of MeSH terms relative to all terms in queries was 18.5% (13.7-25.5).

The V1 queries returned a total of 206,095 items, of which 1628 (0.79%) were included in the GS (Table 1). The V2 queries returned a total of 157,698 items, of which 1473 (0.93%) were included in the GS. In other words, an average of 820.29 additional articles per SR had to be screened for V1, relative to V2. Furthermore, V1 retrieved an average of 2.62 additional relevant articles, when compared with V2.

**Figure 3:**
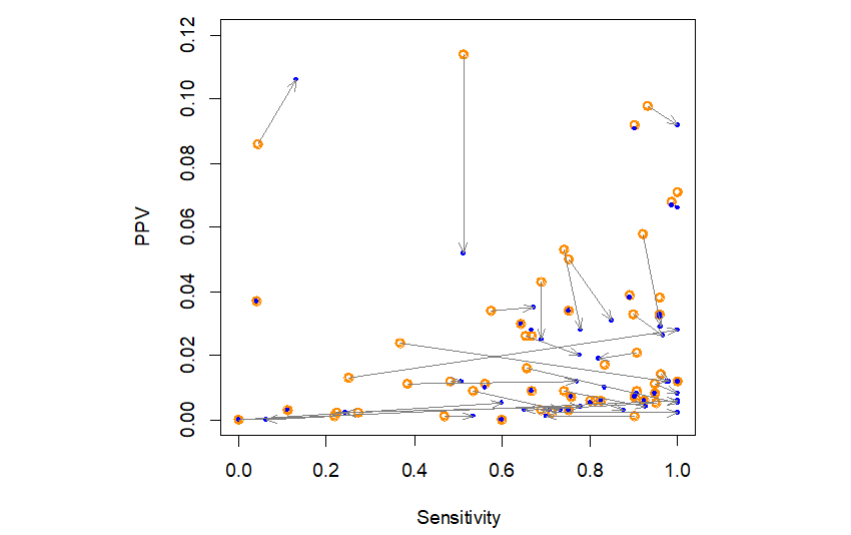
Contribution of Medical Subject Headings (MeSH) terms to the queries. The orange circles correspond to V2 (free-text terms only), and the blue dots correspond to V1 (free-text terms and MeSH terms). PPV: positive predictive value.

The median (Q1-Q3) sensitivities of queries V1 and V2 were 77.8% (62.1%-95.2%) and 71.4% (42.6%-90%), respectively (Table 2). The median (Q1-Q3) PPV of queries V1 and V2 were 0.9% (0.3%-2.8%) and 1.1% (0.3%-3.4%), respectively. The median (Q1-Q3) *F*_1_-scores of queries V1 and V2 were 1.8% (0.7%-5.4%) and 2.2% (0.7%-6.1%), respectively. A graphic visualization of the sensitivity and PPV per SR showed that the addition of MeSH terms to a query typically increased the sensitivity but decreased the PPV (Figure 3). Furthermore, it can be seen that the transition from V2 to V1 had no effect for many SRs.

**Table 2:** Comparison of the performance levels of queries V1, V2, and V3.

Query	Sensitivity, median (IQR)	PPV^a^, median (IQR)	*F*_1_-score, median (IQR)	Number of results, median (IQR)	Number of GS^b^ items found, median (IQR)	Number of results per GS item found, median (IQR)
Query V1 (MeSH^c^ and FTTs^d^)	77.8 (62.1-95.2)	0.9 (0.3-2.8)	1.8 (0.7-5.4)	1950 (657.50-6167.00)	17 (7.00-36.50)	108.857 (35.062-298.574)
Query V2 (FTTs only)	71.4 (42.6-90)	1.1 (0.3-3.4)	2.2 (0.7-6.1)	1166 (301.50-2953.00)	15 (4.50-32.50)	88.667 (29.682-314.848)
Query V3 (MeSH only)	35.7 (0-61.7)	0.5 (0-2.6)	1 (0-3.9)	456 (31.50-2188.50)	4 (0-22.50)	81.305 (20.99-564.125)

^a^PPV: positive predictive value.

^b^GS: gold standard.

^c^MeSH: Medical Subject Headings.

^d^FTT: free-text terms (n=59).

**Figure 4:**
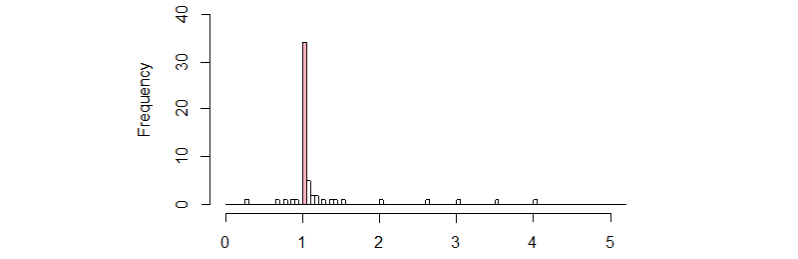
Distribution of the ratio between the number of relevant articles found by V1 and the number found by V2. The pink bar corresponds to the interval (1-1.05). For 2 cases, the ratio corresponded to the division of 0 by 0, and we considered that the result was 1. For other 2 cases, the result of the ratio corresponded to infinity (division by 0).

Overall, V1 provided 8.49% more of the GS’s items than V2 and 35.55% more of the GS’s items than V3. V2 provided 27.06% more of the GS’s items than V3. The ratio between the number of GS references retrieved by V1 and the number retrieved by V2 was within the interval (0-1.05) in 66% (39/59) cases (Figure 4). In 59% (35/59) of cases, the ratio was 1 or less. In other words, the transition from V1 to V2 did not have a marked effect on the number of relevant articles retrieved for more than half of the SRs. 

**Figure 5:**
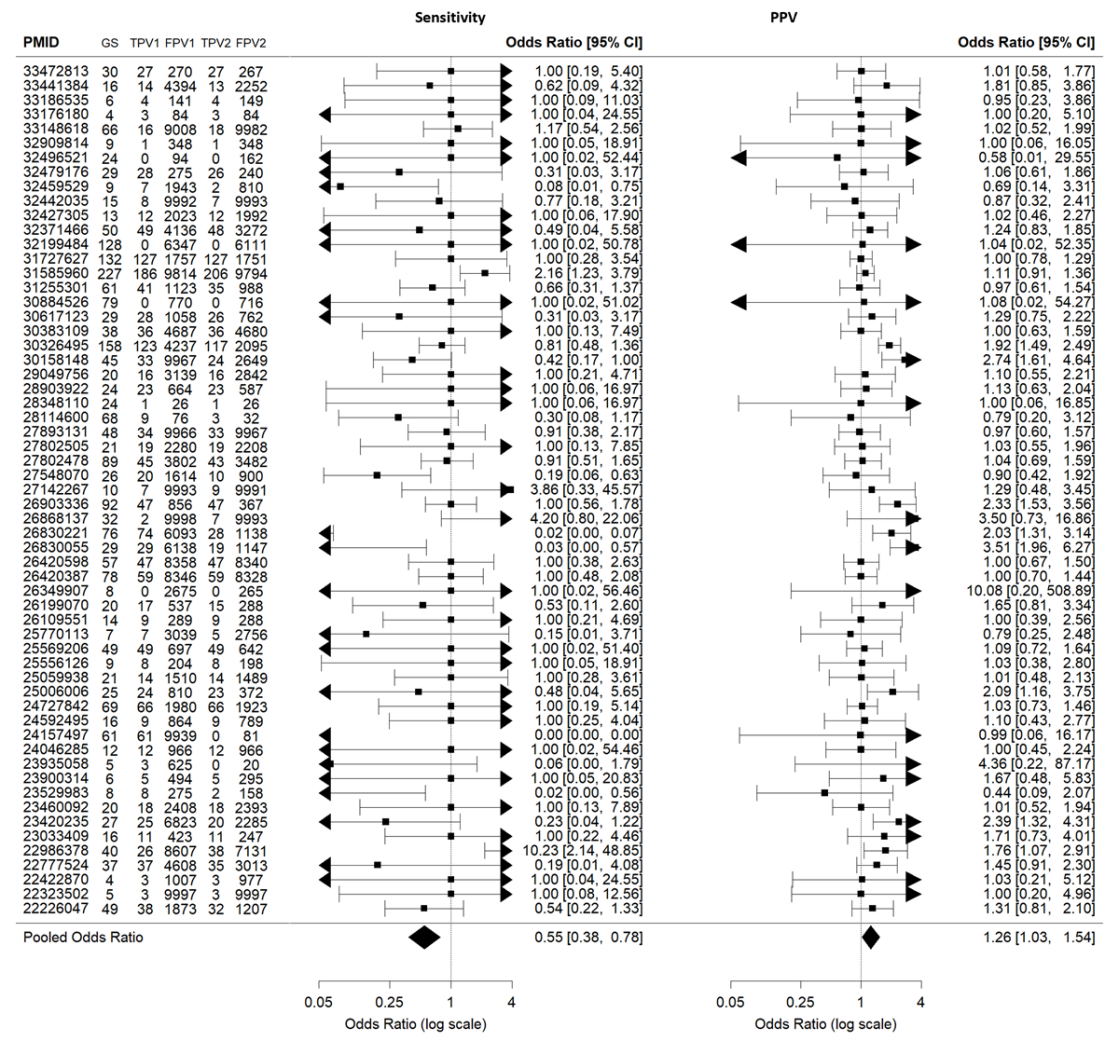
Forest plot of the odds ratio (OR) for V2 versus V1. An OR>1 means that V2 was better than V1 and so that inclusion of the Medical Subject Headings (MeSH) terms was harmful. An OR<1 means that V2 was worse than V1 and so that MeSH terms were useful. FPV1: false positive V1; FPV2: false positive V2; GS: gold standard; PMID: PubMed Identifier; PPV: positive predictive value; TPV1: true positive V1; TPV2: true positive V2.

We also calculated the ORs for the number of relevant articles retrieved by V2 relative to V1 (Figure 5 [26,31-88]). Overall, the OR (95% CI) for V2 versus V1 was 0.55 (0.38-0.78) for sensitivity and 1.26 (1.03-1.54) for the PPV (Figure 5). The OR (95% CI) for V3 versus V1 was 0.31 (0.23-0.41) for sensitivity and 3.11 (2.15-4.48) for the PPV (Multimedia Appendix 3 [26,31-88]).

## Discussion

### Key Results

The objective of this work was to quantify the utility of MeSH terms in SR queries. To this end, we retrieved the queries drafted by the authors of 59 SRs published in 4 prestigious medical journals. We then modified the V1 query to give a free-text terms only query and a MeSH-only query. Finally, we calculated the 3 queries’ sensitivities, PPVs, and *F*_1_-scores.

Our first key observation was that MeSH terms typically accounted for a nonnegligible proportion (on average, 20.4%) of the terms in the query. Second, the removal of MeSH terms from SR queries decreased the sensitivity (by 6.4%, on the median) and increased the PPV (by 0.2%, on the median). In other words, queries containing both MeSH terms and free-text terms yield an average of 2.62 additional relevant papers per SR, necessitating the screening of an additional 820.29 papers. The cost of screening an additional collected paper was therefore 313.09, which was slightly more than triple the mean reading cost associated with free-text terms only queries (88.67). Third, our results indicated that the deletion of MeSH terms had no effect on the number of relevant articles retrieved for 35 (59%) of the 59 reviews.

### Discussion of the Literature Data

The results of a previous study were similar to those found here; 95% of the relevant articles were retrieved in 67% (49/73) of the analyzed SRs when the query contained free-text terms alone (relative to the V1 query with a mixture of MeSH terms and free-text terms) [22]. Another study with a similar objective gave significantly different results; the free-text terms–only query was 25% less sensitive than MeSH-only query [15]. However, it should be noted that (1) the latter findings were based on a single query, and (2) the MeSH terms were converted to free-text terms manually, with a relatively limited set of synonyms used in the free-text terms strategy.

Furthermore, 3 messages should be highlighted. First, MeSH terms remain an indispensable tool for SRs despite the significant advancements in free-text search engines, especially in an era where the quality of SRs is declining [89]. Second, free-text terms appear to contribute more effectively to the retrieval of relevant articles compared with MeSH terms. Third, mixed queries (combining free-text and MeSH terms) exhibit poor PPV; for rapid literature reviews, it is preferable to use either MeSH terms or free-text terms exclusively.

Our study involved queries developed by experienced researchers; choosing free-text terms can be challenging and requires expertise. It is possible that clinicians with limited experience in literature searching struggle to choose free-text terms effectively, and yet, bibliographic research among clinicians is essential [90]. MeSH terms offer a distinct advantage over free-text terms by covering a broad range of vocabulary, which can be particularly beneficial for clinicians, early-career researchers, or nonnative English speakers. In such cases, incorporating MeSH terms can help clinicians construct more comprehensive and effective queries.

### Discussion of the Method

The GS comprised solely MEDLINE-indexed documents with a PMID. This choice was restrictive but technically essential, given that the 3 queries were submitted to the PubMed search engine. However, our restriction to documents with a PMID increased the queries’ sensitivities and decreased their PPVs. We expect this bias to be nondifferential, insofar as it should affect the 3 types of queries in the same way.

The publications with PMIDs 26420387 [38] and 26420598 [83] were written by the same authors and were based on the same search query. This was also the case for PMIDs 26830055 [45] and 26830221 [82]. However, we considered these publications to be independent SRs, insofar as the corresponding GSs were different.

### Strengths and Weaknesses

#### Strengths

One strength of our study is that we used queries from a number of different researchers and research centers; this should mean that our results are more representative of currently used search strategies. Furthermore, the automatic transformation of V1 to V2 probably helped us to avoid any bias associated with the differences in an individual’s knowledge of the MeSH thesaurus.

#### Weaknesses

Interpreting the results of V3 is delicate because the authors’ queries are not designed to remain viable when ignoring all [tiab] and [all fields], etc. Indeed, after transformation to V3, a total of 11 queries become nonviable and return zero items.

In addition, it is important to note that the use of MeSH terms by the authors of the included SRs may be suboptimal and depends on each author’s level of expertise. We assessed the quality of the MeSH selected by the authors of the included SR, not the actual utility of the MeSH as a feature. Finally, we are not able to measure the free-text terms retrieved from initial PubMed searches using only MeSH terms. However, the initial queries using MeSH terms alone may have enriched the search by helping to identify relevant free-text terms. It represents a potentially valuable contribution of MeSH terms that we do not measure here.

### Perspectives

Our results and the literature data provide quantitative information on the use and value of MeSH terms in the queries used for SRs. MeSH terms still appear to be important for achieving a comprehensive SR. Our results also emphasized how difficult it is to build a query for an SR and highlighted the significant variability in the results obtained; the search strategies are a matter of concern for researchers [91-93]. With a view to gaining insights into the possible benefits of MeSH terms for use by less experienced researchers, it would be interesting to conduct a similar study of literature searches performed by clinicians. Finally, our study also highlights that any bibliographic research involves a tedious process of sifting through articles, akin to finding a needle in a haystack. While the authors of SRs perform this task efficiently, inexperienced clinicians might find it discouraging to search for scientific articles. New tools based on network analysis [94] could help these clinicians find relevant articles more quickly.

### Conclusion

The objective of this study was to estimate the utility of MeSH terms, selected by authors, in SR queries by analyzing the queries from 59 SRs published in 4 high-impact medical journals in general medicine. Our results revealed that removing MeSH terms from a query decreases sensitivity while slightly increasing the PPV. Queries containing both MeSH and free-text terms yielded more relevant articles but required screening many additional papers. Despite this additional workload, MeSH terms remain indispensable for SRs and can be particularly beneficial for inexperienced clinicians or nonnative English speakers, aiding in constructing more comprehensive queries. However, mixed queries combining MeSH and free-text terms show poor PPV, suggesting the exclusive use of either MeSH terms or free-text terms for rapid reviews.

## Appendix

Multimedia Appendix 1 List of literature reviews analyzed.

Multimedia Appendix 2 All systematic reviews' queries: V1, V2, and V3.

Multimedia Appendix 3 Forest plot of the OR for V3 versus V1. An OR>1 means that V3 was better than V1. FPV1: false positive V1; FPV2: false positive V2; GS: gold standard; PMID: PubMed Identifier; OR: odds ratio; PPV: positive predictive value; TPV1: true positive V1; TPV2: true positive V2.

## Abbreviations

ATM: automatic term mapping
GS: gold standard
MeSH: Medical Subject Headings
NLM: US National Library of Medicine
OR: odds ratio
PMID: PubMed Identifier
PPV: positive predictive value
SR: systematic review
V1: original query
V2: query with free-text terms only
V3: query with MeSH terms only
