# Supplementary material for: Added Value of Medical Subject Headings Terms in Search Strategies of Systematic Reviews: Comparative Study
Source: J Med Internet Res. 2024 Nov 19;26:e53781. doi: 10.2196/53781 (PMC11615561; doi:10.2196/53781)
Supplement: Multimedia Appendix 1 [file jmir_v26i1e53781_app1.docx]

**List of literature reviews analyzed**

PMID:[24592495] Anglemyer A, Horvath T, Rutherford G. The accessibility of firearms and risk for suicide and homicide victimization among household members: a systematic review and meta-analysis. Ann Intern Med 2014;160:101–10.

PMID:[22323502] Asbridge M, Hayden JA, Cartwright JL. Acute cannabis consumption and motor vehicle collision risk: systematic review of observational studies and meta-analysis. BMJ 2012;344:e536.

PMID:[30884526] Balk EM, Rofeberg VN, Adam GP, Kimmel HJ, Trikalinos TA, Jeppson PC. Pharmacologic and Nonpharmacologic Treatments for Urinary Incontinence in Women: A Systematic Review and Network Meta-analysis of Clinical Outcomes. Ann Intern Med 2019;170:465–79.

PMID:[26868137] Bangalore S, Fakheri R, Toklu B, Messerli FH. Diabetes mellitus as a compelling indication for use of renin angiotensin system blockers: systematic review and meta-analysis of randomized trials. BMJ 2016;352:i438.

PMID:[28903922] Batelaan NM, Bosman RC, Muntingh A, Scholten WD, Huijbregts KM, van Balkom AJLM. Risk of relapse after antidepressant discontinuation in anxiety disorders, obsessive-compulsive disorder, and post-traumatic stress disorder: systematic review and meta-analysis of relapse prevention trials. BMJ 2017;358:j3927.

PMID:[31585960] Bellou V, Belbasis L, Konstantinidis AK, Tzoulaki I, Evangelou E. Prognostic models for outcome prediction in patients with chronic obstructive pulmonary disease: systematic review and critical appraisal. BMJ 2019;367:l5358.

PMID:[23420235] Boland MV, Ervin AM, Friedman DS, Jampel HD, Hawkins BS, Vollenweider D, et al. Comparative effectiveness of treatments for open-angle glaucoma: a systematic review for the U.S. Preventive Services Task Force. Ann Intern Med 2013;158:271–9.

PMID:[26420387] Bolland MJ, Leung W, Tai V, Bastin S, Gamble GD, Grey A, et al. Calcium intake and risk of fracture: systematic review. BMJ 2015;351:h4580.

PMID:[25569206] Carter JL, Coletti RJ, Harris RP. Quantifying and monitoring overdiagnosis in cancer screening: a systematic review of methods. BMJ 2015;350:g7773.

PMID:[33148618] Chersich MF, Pham MD, Areal A, Haghighi MM, Manyuchi A, Swift CP, et al. Associations between high temperatures in pregnancy and risk of preterm birth, low birth weight, and stillbirths: systematic review and meta-analysis. BMJ 2020;371:m3811.

PMID:[30158148] Chowdhury R, Ramond A, O’Keeffe LM, Shahzad S, Kunutsor SK, Muka T, et al. Environmental toxic metal contaminants and risk of cardiovascular disease: systematic review and meta-analysis. BMJ 2018;362:k3310.

PMID:[25770113] Conway R, Low C, Coughlan RJ, O’Donnell MJ, Carey JJ. Methotrexate use and risk of lung disease in psoriasis, psoriatic arthritis, and inflammatory bowel disease: systematic literature review and meta-analysis of randomised controlled trials. BMJ 2015;350:h1269.

PMID:[31255301] Drolet M, nard É, rez N, Brisson M, Ali H, Boily MC, et al. Population-level impact and herd effects following the introduction of human papillomavirus vaccination programmes: updated systematic review and meta-analysis. Lancet 2019;394:497–509.

PMID:[22226047] Edmond KM, Kortsalioudaki C, Scott S, Schrag SJ, Zaidi AK, Cousens S, et al. Group B streptococcal disease in infants aged younger than 3 months: systematic review and meta-analysis. Lancet 2012;379:547–56.

PMID:[26830055] Eng J, Wilson RF, Subramaniam RM, Zhang A, Suarez-Cuervo C, Turban S, et al. Comparative Effect of Contrast Media Type on the Incidence of Contrast-Induced Nephropathy: A Systematic Review and Meta-analysis. Ann Intern Med 2016;164:417–24.

PMID:[33472813] Ferreira GE, McLachlan AJ, Lin CC, Zadro JR, Abdel-Shaheed C, O’Keeffe M, et al. Efficacy and safety of antidepressants for the treatment of back pain and osteoarthritis: systematic review and meta-analysis. BMJ 2021;372:m4825.

PMID:[32496521] Ferreyro BL, Angriman F, Munshi L, Del Sorbo L, Ferguson ND, Rochwerg B, et al. Association of Noninvasive Oxygenation Strategies With All-Cause Mortality in Adults With Acute Hypoxemic Respiratory Failure: A Systematic Review and Meta-analysis. JAMA 2020;324:57–67.

PMID:[24046285] Gagne JJ, Bykov K, Choudhry NK, Toomey TJ, Connolly JG, Avorn J. Effect of smoking on comparative efficacy of antiplatelet agents: systematic review, meta-analysis, and indirect comparison. BMJ 2013;347:f5307.

PMID:[33186535] Gencer B, Marston NA, Im K, Cannon CP, Sever P, Keech A, et al. Efficacy and safety of lowering LDL cholesterol in older patients: a systematic review and meta-analysis of randomised controlled trials. Lancet 2020;396:1637–43.

PMID:[33441384] Goldenberg JZ, Day A, Brinkworth GD, Sato J, Yamada S, nsson T, et al. Efficacy and safety of low and very low carbohydrate diets for type 2 diabetes remission: systematic review and meta-analysis of published and unpublished randomized trial data. BMJ 2021;372:m4743.

PMID:[23900314] Goto A, Arah OA, Goto M, Terauchi Y, Noda M. Severe hypoglycaemia and cardiovascular disease: systematic review and meta-analysis with bias analysis. BMJ 2013;347:f4533.

PMID:[25006006] Haycock PC, Heydon EE, Kaptoge S, Butterworth AS, Thompson A, Willeit P. Leucocyte telomere length and risk of cardiovascular disease: systematic review and meta-analysis. BMJ 2014;349:g4227.

PMID:[26349907] Hendriksen JM, Geersing GJ, Lucassen WA, Erkens PM, Stoffers HE, van Weert HC, et al. Diagnostic prediction models for suspected pulmonary embolism: systematic review and independent external validation in primary care. BMJ 2015;351:h4438.

PMID:[32459529] Hernandez AV, Roman YM, Pasupuleti V, Barboza JJ, White CM. Hydroxychloroquine or Chloroquine for Treatment or Prophylaxis of COVID-19: A Living Systematic Review. Ann Intern Med 2020;173:287–96.

PMID:[22422870] Hu EA, Pan A, Malik V, Sun Q. White rice consumption and risk of type 2 diabetes: meta-analysis and systematic review. BMJ 2012;344:e1454.

PMID:[31727627] Huang SW, Tsai CY, Tseng CS, Shih MC, Yeh YC, Chien KL, et al. Comparative efficacy and safety of new surgical treatments for benign prostatic hyperplasia: systematic review and network meta-analysis. BMJ 2019;367:l5919.

PMID:[32427305] Hughes D, Judge C, Murphy R, Loughlin E, Costello M, Whiteley W, et al. Association of Blood Pressure Lowering With Incident Dementia or Cognitive Impairment: A Systematic Review and Meta-analysis. JAMA 2020;323:1934–44.

PMID:[26199070] Imamura F, O’Connor L, Ye Z, Mursu J, Hayashino Y, Bhupathiraju SN, et al. Consumption of sugar sweetened beverages, artificially sweetened beverages, and fruit juice and incidence of type 2 diabetes: systematic review, meta-analysis, and estimation of population attributable fraction. BMJ 2015;351:h3576.

PMID:[32909814] Juraschek SP, Hu JR, Cluett JL, Ishak A, Mita C, Lipsitz LA, et al. Effects of Intensive Blood Pressure Treatment on Orthostatic Hypotension : A Systematic Review and Individual Participant-based Meta-analysis. Ann Intern Med 2021;174:58–68.

PMID:[27893131] Kavalieratos D, Corbelli J, Zhang D, Dionne-Odom JN, Ernecoff NC, Hanmer J, et al. Association Between Palliative Care and Patient and Caregiver Outcomes: A Systematic Review and Meta-analysis. JAMA 2016;316:2104–14.

PMID:[25059938] Kerrigan D, Kennedy CE, Morgan-Thomas R, Reza-Paul S, Mwangi P, Win KT, et al. A community empowerment approach to the HIV response among sex workers: effectiveness, challenges, and considerations for implementation and scale-up. Lancet 2015;385:172–85.

PMID:[24727842] Kim CA, Rasania SP, Afilalo J, Popma JJ, Lipsitz LA, Kim DH. Functional status and quality of life after transcatheter aortic valve replacement: a systematic review. Ann Intern Med 2014;160:243–54.

PMID:[27548070] Kim DH, Kim CA, Placide S, Lipsitz LA, Marcantonio ER. Preoperative Frailty Assessment and Outcomes at 6 Months or Later in Older Adults Undergoing Cardiac Surgical Procedures: A Systematic Review. Ann Intern Med 2016;165:650–60.

PMID:[32371466] Kisely S, Warren N, McMahon L, Dalais C, Henry I, Siskind D. Occurrence, prevention, and management of the psychological effects of emerging virus outbreaks on healthcare workers: rapid review and meta-analysis. BMJ 2020;369:m1642.

PMID:[23529983] Kramer CK, Zinman B, Gross JL, Canani LH, Rodrigues TC, Azevedo MJ, et al. Coronary artery calcium score prediction of all cause mortality and cardiovascular events in people with type 2 diabetes: systematic review and meta-analysis. BMJ 2013;346:f1654.

PMID:[26109551] Lamont K, Scott NW, Jones GT, Bhattacharya S. Risk of recurrent stillbirth: systematic review and meta-analysis. BMJ 2015;350:h3080.

PMID:[25556126] Liao WC, Tu YK, Wu MS, Lin JT, Wang HP, Chien KL. Blood glucose concentration and risk of pancreatic cancer: systematic review and dose-response meta-analysis. BMJ 2015;350:g7371.

PMID:[32479176] Lyles CR, Nelson EC, Frampton S, Dykes PC, Cemballi AG, Sarkar U. Using Electronic Health Record Portals to Improve Patient Engagement: Research Priorities and Best Practices. Ann Intern Med 2020;172:S123–9.

PMID:[23935058] m N, Enebrink P, n EM, Lindblom J, ö S, Hanson RK. Preventing sexual abusers of children from reoffending: systematic review of medical and psychological interventions. BMJ 2013;347:f4630.

PMID:[32199484] Martinez L, Cords O, Horsburgh CR, Andrews JR, Acuna-Villaorduna C, Desai Ahuja S, et al. The risk of tuberculosis in children after close exposure: a systematic review and individual-participant meta-analysis. Lancet 2020;395:973–84.

PMID:[28114600] Mendelson A, Kondo K, Damberg C, Low A, apuaka M, Freeman M, et al. The Effects of Pay-for-Performance Programs on Health, Health Care Use, and Processes of Care: A Systematic Review. Ann Intern Med 2017;166:341–53.

PMID:[30383109] Naik RP, Smith-Whitley K, Hassell KL, Umeh NI, de Montalembert M, Sahota P, et al. Clinical Outcomes Associated With Sickle Cell Trait: A Systematic Review. Ann Intern Med 2018;169:619–27.

PMID:[32442035] nemann HJ, Khabsa J, Solo K, Khamis AM, Brignardello-Petersen R, El-Harakeh A, et al. Ventilation Techniques and Risk for Transmission of Coronavirus Disease, Including COVID-19: A Living Systematic Review of Multiple Streams of Evidence. Ann Intern Med 2020;173:204–16.

PMID:[27802505] Newberry SJ, FitzGerald JD, Motala A, Booth M, Maglione MA, Han D, et al. Diagnosis of Gout: A Systematic Review in Support of an American College of Physicians Clinical Practice Guideline. Ann Intern Med 2017;166:27–36.

PMID:[30617123] Ochen Y, Beks RB, van Heijl M, Hietbrink F, Leenen LPH, van der Velde D, et al. Operative treatment versus nonoperative treatment of Achilles tendon ruptures: systematic review and meta-analysis. BMJ 2019;364:k5120.

PMID:[22986378] Prvu Bettger J, Alexander KP, Dolor RJ, Olson DM, Kendrick AS, Wing L, et al. Transitional care after hospitalization for acute stroke or myocardial infarction: a systematic review. Ann Intern Med 2012;157:407–16.

PMID:[30326495] Rotenstein LS, Torre M, Ramos MA, Rosales RC, Guille C, Sen S, et al. Prevalence of Burnout Among Physicians: A Systematic Review. JAMA 2018;320:1131–50.

PMID:[27142267] Salvo F, Moore N, Arnaud M, Robinson P, Raschi E, De Ponti F, et al. Addition of dipeptidyl peptidase-4 inhibitors to sulphonylureas and risk of hypoglycaemia: systematic review and meta-analysis. BMJ 2016;353:i2231.

PMID:[28348110] Schandelmaier S, Kaushal A, Lytvyn L, Heels-Ansdell D, Siemieniuk RA, Agoritsas T, et al. Low intensity pulsed ultrasound for bone healing: systematic review of randomized controlled trials. BMJ 2017;356:j656.

PMID:[29049756] Selby K, Baumgartner C, Levin TR, Doubeni CA, Zauber AG, Schottinger J, et al. Interventions to Improve Follow-up of Positive Results on Fecal Blood Tests: A Systematic Review. Ann Intern Med 2017;167:565–75.

PMID:[26903336] Shankar-Hari M, Phillips GS, Levy ML, Seymour CW, Liu VX, Deutschman CS, et al. Developing a New Definition and Assessing New Clinical Criteria for Septic Shock: For the Third International Consensus Definitions for Sepsis and Septic Shock (Sepsis-3). JAMA 2016;315:775–87.

PMID:[27802478] Shekelle PG, Newberry SJ, FitzGerald JD, Motala A, O’Hanlon CE, Tariq A, et al. Management of Gout: A Systematic Review in Support of an American College of Physicians Clinical Practice Guideline. Ann Intern Med 2017;166:37–51.

PMID:[26830221] Subramaniam RM, Suarez-Cuervo C, Wilson RF, Turban S, Zhang A, Sherrod C, et al. Effectiveness of Prevention Strategies for Contrast-Induced Nephropathy: A Systematic Review and Meta-analysis. Ann Intern Med 2016;164:406–16.

PMID:[26420598] Tai V, Leung W, Grey A, Reid IR, Bolland MJ. Calcium intake and bone mineral density: systematic review and meta-analysis. BMJ 2015;351:h4183.

PMID:[33176180] Thomalla G, Boutitie F, Ma H, Koga M, Ringleb P, Schwamm LH, et al. Intravenous alteplase for stroke with unknown time of onset guided by advanced imaging: systematic review and meta-analysis of individual patient data. Lancet 2020;396:1574–84.

PMID:[23460092] Weaver SJ, Lubomksi LH, Wilson RF, Pfoh ER, Martinez KA, Dy SM. Promoting a culture of safety as a patient safety strategy: a systematic review. Ann Intern Med 2013;158:369–74.

PMID:[23033409] Wehner MR, Shive ML, Chren MM, Han J, Qureshi AA, Linos E. Indoor tanning and non-melanoma skin cancer: systematic review and meta-analysis. BMJ 2012;345:e5909.

PMID:[24157497] Wu HY, Huang JW, Lin HJ, Liao WC, Peng YS, Hung KY, et al. Comparative effectiveness of renin-angiotensin system blockers and other antihypertensive drugs in patients with diabetes: systematic review and bayesian network meta-analysis. BMJ 2013;347:f6008.

PMID:[22777524] Yeh HC, Brown TT, Maruthur N, Ranasinghe P, Berger Z, Suh YD, et al. Comparative effectiveness and safety of methods of insulin delivery and glucose monitoring for diabetes mellitus: a systematic review and meta-analysis. Ann Intern Med 2012;157:336–47.
