# Supplementary figures and images for: Added Value of Medical Subject Headings Terms in Search Strategies of Systematic Reviews: Comparative Study
Source: J Med Internet Res. 2024 Nov 19;26:e53781. doi: 10.2196/53781 (PMC11615561; doi:10.2196/53781)

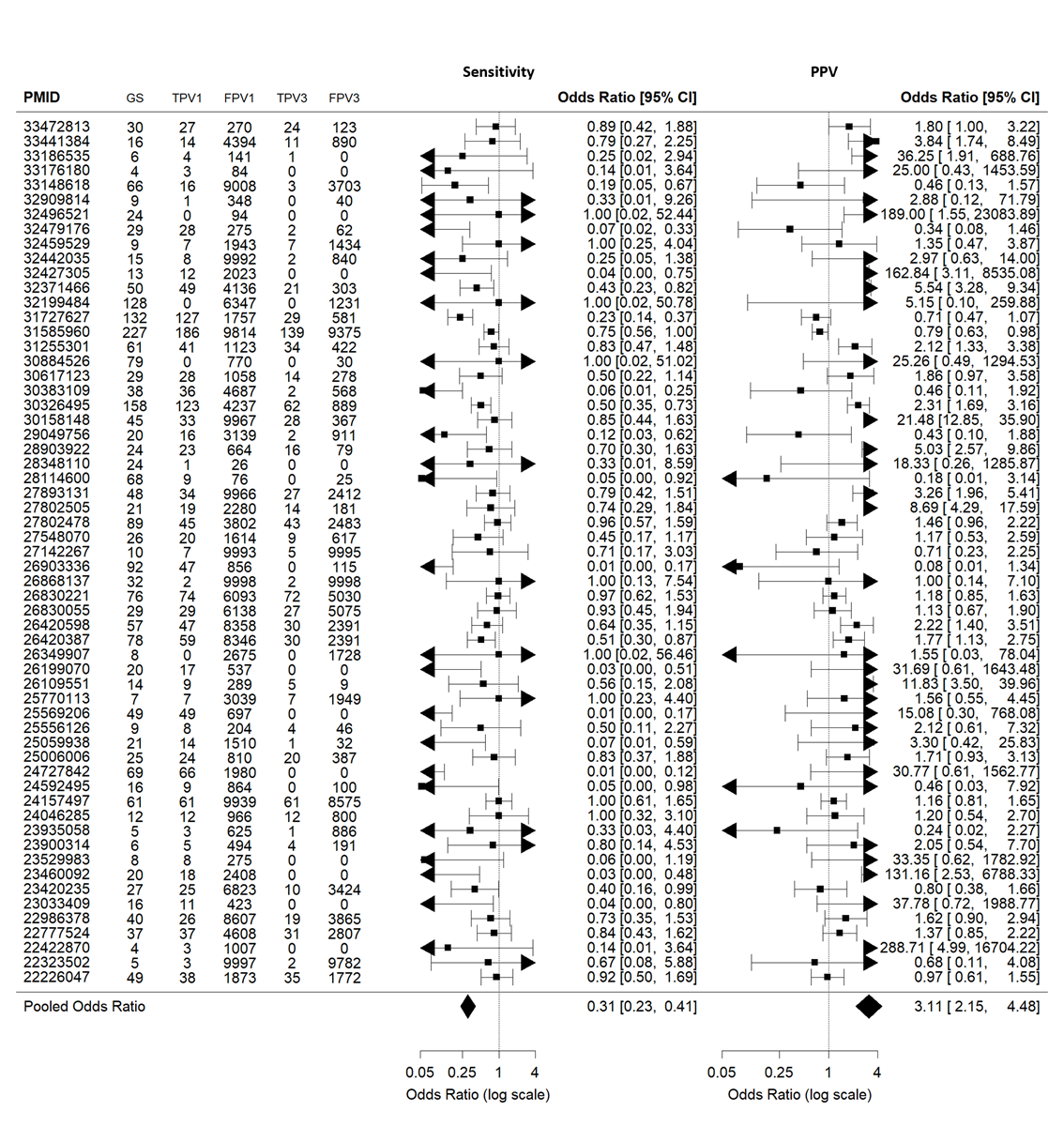

Supplement: Multimedia Appendix 3 [file jmir_v26i1e53781_app3.png]
